# The Effect of *Buddleja officinalis* Maxim Eye Drops on Morphology and Apoptosis in Lacrimal Gland of Experimental Dry Eye Rabbit Model

**DOI:** 10.1155/2019/5916243

**Published:** 2019-02-05

**Authors:** Genyan Qin, Yasha Zhou, Jun Peng, Youwei Zhang, Xiaofang Peng, Qinghua Peng, Yijing Yang

**Affiliations:** ^1^The Domestic First-class Discipline Construction Project of Chinese Medicine, Hunan University of Chinese Medicine, Changsha, Hunan Province 410208, China; ^2^Department of Ophthalmology, No. 1 Traditional Chinese Medicine Hospital in Changde, Changde, Hunan Province 415000, China; ^3^Department of Ophthalmology, The First Hospital, Hunan University of Chinese Medicine, Changsha, Hunan Province 410007, China

## Abstract

The purpose of this study was to investigate the effects of *Buddleja officinalis* Maxim eye drops on morphology and apoptosis in lacrimal glands of the experimental dry eye rabbit model. A total of thirty-six male rabbits were divided into six study groups, consisting of the control group and the dry eye rabbit model group (without any treatment), the dry eye rabbit model group treated with testosterone, and the dry eye rabbit model group treated with different concentrations of *Buddleja officinalis* Maxim eye drops (1.0 mg/ml, 1.5 mg/ml and 3.0 mg/ml). The lacrimal glands were evaluated by hematoxylin-eosin staining and immunohistochemistry. *Buddleja officinalis* Maxim eye drops can improve the morphological structure of the lacrimal gland in the dry eye model of castrated rabbits. The average optical density values of PI3K, Akt, and caspase-9 protein in the lacrimal gland tissue of the 3 mg/ml *Buddleja officinalis* Maxim eye drops group were significantly different from those in the model group (*P* < 0.01) and similar to the testosterone control group and the control group (*P* > 0.05). *Buddleja officinalis* Maxim eye drops can improve the morphological structure of the lacrimal gland in the dry eye model of castrated rabbits.

## 1. Introduction

Dry eye disease (DED) is a multifactorial disease of the ocular surface characterized by a loss of homeostasis of the tear film and accompanied by ocular symptoms, in which tear film instability and hyperosmolarity, ocular surface inflammation and damage, and neurosensory abnormalities play etiological roles [1]. At present, it has become the most common eye disease except refractive error. Some studies have confirmed that decreased androgen levels are an important factor in the dry eye [2].

The basic treatment principle of the dry eye is to improve the symptoms of eye discomfort and protect the patient's visual function, by supplementing or restoring the normal components of tears, restoring the normal anatomy of the surface of the eye, inhibiting the inflammation of the surface of the eye, and finally recovering the ocular surface and the tear film, so as to reach the normal anatomy and physiological function [3]. Currently, we used treatment methods including physical therapy, intense pulsed light (IPL), moisture chamber glasses, artificial tears, lipid replacement therapy, anti-inflammatory therapy, and lacrimal duct embolism [4], but the efficacy is not satisfactory, and even caused many side effects. Therefore, Chinese medicine with abundant natural medicine resources has broad prospects in the field of dry eye treatment and has great potential for discovering innovative drugs.

*Buddleja officinalis* Maxim is a kind plant of the genus *Buddleia*, and we can extract 8 flavonoids from its flower buds, which is a type of phytohormone. Flavonoids can bind to the androgen receptor to act as an androgenic activity because it has the same chemical structure as androgen-heterocyclic polyphenols and to maintain the normal level of androgen in the body to treat some diseases caused by decreased androgen levels [5]. Our previous experimental study confirmed that *Buddleja officinalis* Maxim granules can downregulate the expression of apoptotic factors Bax, Fas, and FasL in ovariectomized rabbit lacrimal gland cells and upregulate the expression of Bcl-2, thereby inhibiting the apoptosis of lacrimal gland cells and maintaining the basis amount of secretion of the lacrimal gland, but its effect is weaker than androgen [6]. The related preparations of *Buddleja officinalis* Maxim granules and their medicinal herbs have a positive therapeutic effect on the dry eyes caused by decreased androgen levels. Therefore, we suspect that it may also have a certain effect on the treatment of the dry eye caused by decreased sex hormone levels.

This study mainly studied the effects of *Buddleja officinalis* Maxim eye drops on the morphology of the lacrimal gland and the apoptosis of lacrimal gland cells in the castrated rabbit dry eye model, and further explored the exact effect of *Buddleja officinalis* Maxim eye drops on the dry eye and its possible mechanism to seek new treatments of the dry eye.

## 2. Materials and Methods

### 2.1. Animals

There were six rabbit groups (totally thirty-six rabbit, *n*=6 rabbit each), i.e., the control group (male rabbit without any treatment), the dry eye model rabbit group, dry eye rabbit group treated with testosterone, the dry eye rabbit model group treated with 1 mg/ml *Buddleja officinalis* Maxim eye drops, the dry eye rabbit model group treated with 1.5 mg/ml *Buddleja officinalis* Maxim eye drops, and the dry eye rabbit model group treated with 3 mg/ml *Buddleja officinalis* Maxim eye drops. The administration of *Buddleja officinalis* Maxim eye drops was performed for a month.

### 2.2. Dry Eye Model of Rabbit due to Castration

To perform castration, male New Zealand white rabbits were anesthetized using 25% urethane (4 ml/kg). Anesthesia was satisfactory and then fixed on the stent. Testicular area was a disinfected, aseptic surgical procedure. We squeezed one side of the testicle from the abdominal cavity into the scrotum and fixed it and then using a sterile blade to make an incision in the scrotum area, forcefully extruded the testicle and ligatured the spermatic vein and the vas deferens and then removed the testis and epididymis and applied a proper amount of penicillin powder to prevent infection, continuous suturing of scrotal skin tissue after local disinfection. The other side of the testis and epididymis removal method is the same [7, 8].

### 2.3. Drugs

#### 2.3.1. Total Flavonoids and Total Phenylpropanoid Extracts of *Buddleja officinalis* Maxim

Total flavonoids and total phenylpropanoid extracts of *Buddleja officinalis* Maxim are prepared by the Department of Pharmacy, the Second Xiangya Hospital of Central South University. The specific extraction process is as follows: we used 60% ethanol to extract 100 kg *Buddleja officinalis* Maxim twice in a row. We added 10 times the volume of ethanol for the first time and 8 times the volume of ethanol for the second time, combined the ethanol extract, recovered the ethanol under reduced pressure to the taste of no alcohol, treated the concentrate with the ZTC clarifying agent, filtered it, filtered the HPD-100 macroporous resin column, and eluted with different concentrations of ethanol. The 60% ethanol eluate was dried in vacuum and pulverized to obtain 1.5 kg of dry paste.

#### 2.3.2. *Buddleja officinalis* Maxim Eye Drops

*Buddleja officinalis* Maxim eye drops is prepared by the College of Pharmacy of Hunan University of Chinese Medicine, and the extracts of *Buddleja officinalis* Maxim is prepared according to the eye drop preparation process. We dissolved the total flavonoids and total phenylpropanoid extracts of *Buddleja officinalis* Maxim with injection water and prepared them by microporous filtration membrane filtration and sterilizing filtrate. According to the preliminary experimental results of our research group, we decided to select five concentrations (3 mg/10 ml, 5 mg/10 ml, 10 mg/10 ml, 15 mg/10 ml, and 30 mg/10 ml). The eye drops were prepared without any preservatives and stored in a 4–8°C refrigerator for about 1 week. We used the *Buddleja officinalis* Maxim eye drops 3 times a day: about eight o'clock, twelve o'clock, and sixteen o'clock.

#### 2.3.3. Testosterone Propionate Injection

Testosterone propionate injection is produced by Tianjin King York Pharmaceutical Co., Ltd., of China (specification: 1 mL: 25 mg). At present, there is no testosterone eye drops applied to the clinic. In Group F, all experimental animal are injected once in every 3 days to reach and supplement the role of androgen, and 2 mg/kg testosterone propionate injection is injected into the muscles of rabbit thighs (in terms of human and rabbit body surface area [9]).

### 2.4. Schirmer's Test I

Schirmer's test I (SIT) values were measured and recorded in each experimental group before modeling, 8 wk after modeling, and 14 d and 28 d after medication. SIT, according to the instructions, the tear secretion test paper was placed at the junction of the outer and outer 1/3 of the conjunctival sac under the eye; gently close the eyes, remove the filter paper after 5 minutes, and measure the wet length of the filter paper, calculated in millimeters.

### 2.5. Diagnostic Criteria for Dry Eye in China

One of the subjective symptoms such as dryness, foreign body sensation, burning sensation, fatigue, and vision fluctuation, and BUT ≤5s or Schirmer I test (no surface anesthesia) ≤5 mm/5 min can diagnose the dry eye [10].

### 2.6. Experimental Process

The experimental animals were fed adaptively in the laboratory for one week to exclude the animals in poor health. The other animals were randomly divided into 6 groups with 6 animals in each group. Except for the control group, the other 5 groups were made models (bilateral testiculectomy and epididymis excision). The test of tear secretion after two months of feeding proved that the animal model was successfully established, and the drug was given on the first day after the successful establishment of the animal model. The control group without any treatment (Group A), the dry eye model rabbit group without any treatment (Group B), the dry eye rabbit model group treated with 1 mg/ml *Buddleja officinalis* Maxim eye drops (Group C, three times a day), the dry eye rabbit model group treated with 1.5 mg/ml *Buddleja officinalis* Maxim eye drops (Group D, three times a day), the dry eye rabbit model group treated with 3 mg/ml *Buddleja officinalis* Maxim eye drops (Group E, three times a day), dry eye rabbit group treated with testosterone (Group F, once every three days) had continuous administration for one month. At the end of the experiment, the lacrimal gland of the animal was removed.

### 2.7. Removing the Lacrimal Gland

New Zealand white rabbits were anesthetized with 25% urethane (4 ml/kg) via ear vein. Anesthesia was satisfactory and then fixed on the stent to remove the lacrimal glands. The lacrimal gland tissue was fixed in formaldehyde fixative solution and sent to the immunohistochemical test. After the end of the experimental, the animals were sacrificed by air embolism.

### 2.8. Light Microscopic Observation of the Morphology of the Lacrimal Gland

The lacrimal gland was immediately placed in 4% paraformaldehyde, embedded in paraffin, and sectioned with hematoxylin-eosin staining. The lacrimal gland structure of each group of New Zealand white rabbits was observed under light microscope.

### 2.9. Immunohistochemistry Was Used to Detect the Expression of Apoptotic Factors PI3K, AKT, and Caspase-9 in Lacrimal Gland Cells of Castrated Rabbits

After the lacrimal gland specimen is dewaxed and hydrated, it is operated according to the instructions of the immunohistochemistry kit. The expression of caspase-9, Akt, and PI3K protein was observed under the optical microscope, and 12 slices were randomly selected from each group. Five 400-fold fields were randomly observed for each slice. All positive particles in the field of view were photographed and accurately selected. The average optical density value was obtained by Image-pro Plus 6.0 analysis software and used to quantitatively express the degree of immunocytochemical reaction. Judging criteria: the cytoplasm staining of positively labeled cells after immunohistochemistry was brown or dark brown. The image analysis system was used for image analysis and processing. The above detection is done through PV-9000 2-step plus Poly-HRP Anti-Mouse/Rabbit IgG Detection System.

### 2.10. Statistical Analysis

Experimental data analysis was performed using SPSS 25.0 system software. All data of the experimental results were expressed as mean ± standard deviation ($\bar{x}$ ± SD). The multiple sets of comparisons satisfy the normal and the variance homogeneity, and the variance analysis is used. If the normality and homogeneity of variance are not satisfied, the rank sum test is used. *P* < 0.01 is considered to be statistically significant.

## 3. Results

### 3.1. Comparison of SIT Values before and after Modeling in Male Rabbits

Before the animal model was built, the SIT values of each group were normal, and there were no difference between the groups (*P* > 0.05). 8 weeks after modeling, the SIT values of 1.0 mg/ml, 1.5 mg/ml, and 3.0 mg/ml *Buddleja officinalis* Maxim eye drops and the testosterone group were significantly decreased, compared with the control group, and the difference was statistically significant (*P* < 0.01) and reached the diagnostic criteria for the dry eye. 14 days after treatment, the SIT values of the treatment groups increased slightly, compared with the model group, and there was no significant difference (*P* > 0.05). 28 days after treatment, the SIT values of the treatment groups increased significantly, of which 1.5 mg/ml *Buddleja officinalis* Maxim eye drops group was similar to the testosterone group (*P* > 0.05) (Table 1).

### 3.2. Light Microscopic Observation of the Morphology of Lacrimal Gland

After the end of the experiment, the lacrimal gland tissue was stained by HE and then observed under the light microscope.

#### 3.2.1. Control Group

The connective tissue of the lacrimal gland divides the glandular tissue into small leaflets of different sizes. The lacrimal gland has a clear structure, and the acinar and lacrimal gland epithelium are uniform in size, neatly arranged, and normal in morphology.

#### 3.2.2. Model Group

The structure of the lacrimal gland is not clear, the size of acinar and lacrimal gland epithelium is different, and the arrangement is disordered. Many acinar atrophy and fusion are formed and did the vacuoles.

#### 3.2.3. 1.0 mg/ml *Buddleja officinalis* Maxim Eye Drops

The lacrimal gland structure is clear, and the acinar and lacrimal gland epithelium are uniform in size and loosely arranged, showing a small amount of acinar atrophy and vacuolization.

#### 3.2.4. 1.5 mg/ml *Buddleja officinalis* Maxim Eye Drops

The lacrimal gland structure is clear, the acinar and lacrimal gland epithelium are uniform in size and arranged neatly, and a small number of vacuoles are formed.

#### 3.2.5. 3.0 mg/ml *Buddleja officinalis* Maxim Eye Drops

**Figure 1:**
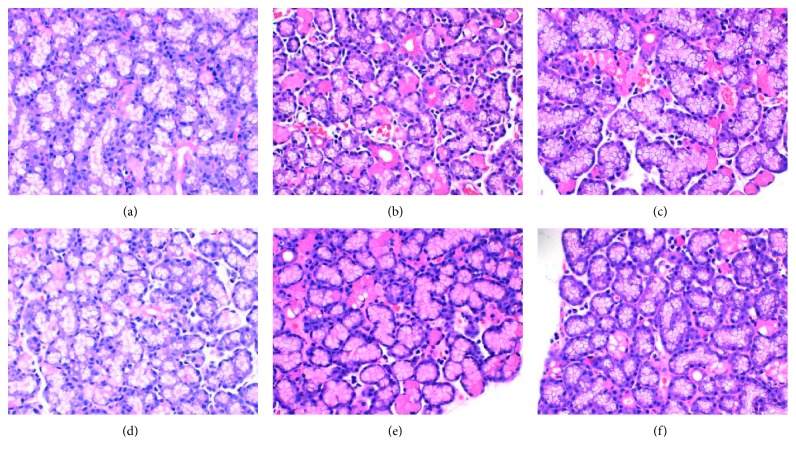
HE staining of the lacrimal gland (×400 times): (a) control group; (b) model group; (c) 1.0 mg/ml *Buddleja officinalis* Maxim eye drops; (d) 1.5 mg/ml *Buddleja officinalis* Maxim eye drops; (e) 3.0 mg/ml *Buddleja officinalis* Maxim eye drops; (f) testosterone group.

The lacrimal gland structure is clear, the acinar and lacrimal gland epithelium are uniform in size and neatly arranged, and the shape is normal, showing a little vacuole (Figure 1).

#### 3.2.6. Testosterone Group

**Figure 2:**
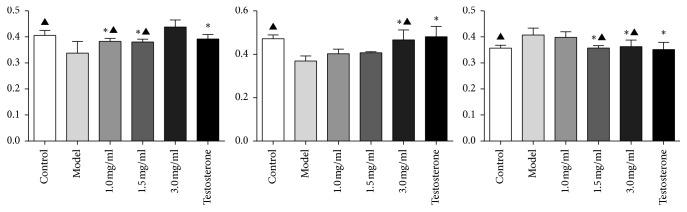
Comparison of average optical density values of (a) PI3K, (b) Akt, and (c) caspase-9.

The lacrimal gland structure is clear, the acinar and lacrimal gland epithelium are uniform in size and neatly arranged, and the shape is normal, showing a little vacuole (Figure 2).

#### 3.2.7. Immunohistochemical Method Was Used to Detect the Expression of Apoptotic Factors PI3K, Akt, and Caspase-9 in Lacrimal Gland of Male Rabbits

Immunohistochemistry was used to detect the expression of the apoptotic factor PI3K in the lacrimal gland of male rabbits. Compared with the testosterone group, there was no statistically significant difference between the control group and 1.0 mg/ml and the 1.5 mg/ml *Buddleja officinalis* Maxim eye drops group (*P* > 0.05). Compared with the control group, there was no significant difference between the 1.0 mg/ml and 1.5 mg/ml *Buddleja officinalis* Maxim eye drops and the testosterone group (*P* > 0.05).

About Akt, compared with the testosterone group, there was no statistically significant difference between the control group and the 3.0 mg/ml *Buddleja officinalis* Maxim eye drops group (*P* > 0.05). Compared with the control group, there was no significant difference between the 3.0 mg/ml *Buddleja officinalis* Maxim eye drops and the testosterone group (*P* > 0.05).

**Figure 3:**
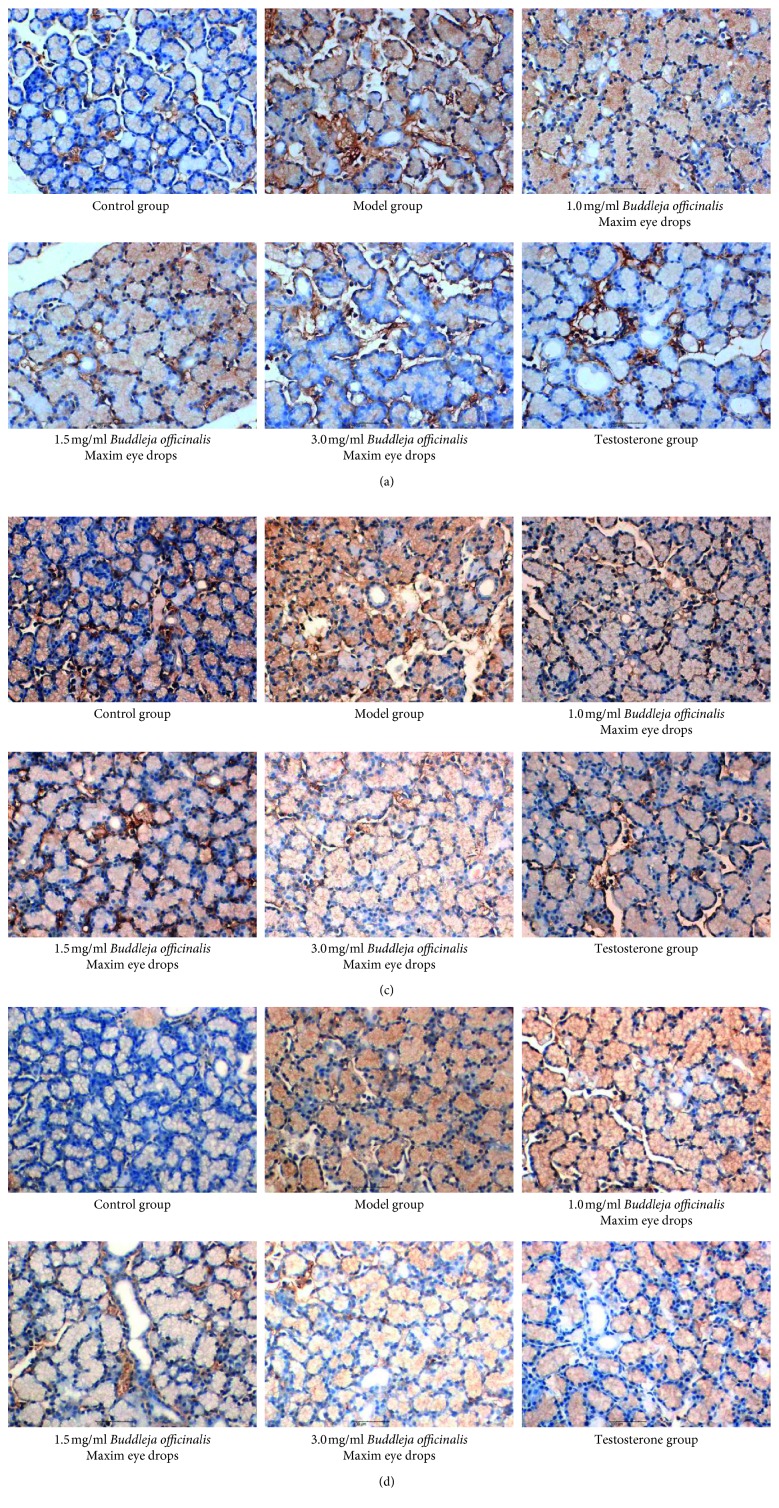
Immunohistochemical observation of (a) PI3K, (b) Akt, and (c) caspase-9 protein expression in the lacrimal gland (×400 times).

About caspase-9, compared with the testosterone group, there was no statistically significant difference between the control group and the 1.5 mg/ml and the 3.0 mg/ml *Buddleja officinalis* Maxim eye drops group (*P* > 0.05). Compared with the control group, there was no significant difference between the 1.5 mg/ml and the 3.0 mg/ml *Buddleja officinalis* Maxim eye drops and the testosterone group (*P* > 0.05) (Table 2; Figures 2 and 3).

## 4. Discussion

Our study found that the use of *Buddleja officinalis* Maxim extract as a dry eye drop improved the lacrimal gland histology and cellular makeup and that this matched the effect of topical testosterone. The average optical density values of PI3K, Akt, and caspase-9 protein in the lacrimal gland tissue of the 3.0 mg/ml *Buddleja officinalis* Maxim eye drops group were significantly different from those in the model group (*P* < 0.01) and like the testosterone control group and the control group (*P* > 0.05).

Dry eye caused by decreased sex hormone levels is a key concern in the dry eye field in recent years. The 2017 TFOS Dry Eye Working Group devoted a discussion on the relationship between biological sex, gender, hormones, and dry eye, and the importance of which is the second only to pathology and treatment [2]. Among them, androgen is a very important position in the regulation of the epidermis and attachment of the eye, which mediates many sex-related differences in the tissue [11]. Related studies have confirmed that the eye is one of the main target organs of sex hormones [12]. Sex hormone receptors (including androgens, estrogens, and progesterone) are widely present in the tear function unit of human, mouse, and rabbit [11]. Among them are the lacrimal gland, meibomian gland, cornea, and conjunctiva. As a target organ of androgen, the lacrimal gland is regulated by androgen levels in its synthesis and secretion [13–15].

In this study, the high-performance liquid chromatography was used to extract the *Buddleja officinalis* Maxim and found that the total flavonoids and phenylpropanoids were the main components of the *Buddleja officinalis* Maxim, and then we made it to eye drops. Eye drop is one of the most commonly used dosage forms for ophthalmic treatment, and the *Buddleja officinalis* Maxim may access to the lacrimal gland through tissue infiltration. Therefore, *Buddleja officinalis* Maxim Eye Drops has a wide range of the application value, but its preparation process and optimal therapeutic concentration need to be further explored. The effective components of *Buddleja officinalis* Maxim also need to be further explored. And in our research, we focused on the screening of drug concentrations, and ignoring the design of the placebo-treated group and the sham-operated group, the absence of placebo-treated and sham-operated groups of rabbits was a limitation in our study, and we will correct it in the future experiments to increase the rigour of the experiment.

## Figures and Tables

**Table 1:** Comparison of SIT values at the same time of each group ($\bar{x}$ ± S).

Group	Before modeling	8 wk after modeling	14 d after medication	28 d after medication
A	15.50 ± 1.246	16.08 ± 0.975^*∗∗*^	17.00 ± 0.862^*∗∗*^	18.42 ± 0.596^*∗∗*^^∆∆^
B	13.83 ± 1.492^▲^	2.92 ± 0.499^▲▲^	3.58 ± 0.557^▲▲^	2.42 ± 0.583^▲▲∆∆^
C	15.50 ± 1.011^▲^^*∗*^	3.42 ± 0.583^▲▲^^*∗*^	6.75 ± 0.808^▲▲^^*∗∗*^	8.92 ± 0.398^▲▲^^*∗∗*^^∆^
D	16.67 ± 1.378^▲^^*∗*^	4.33 ± 0.762^▲▲^^*∗*^	8.25 ± 0.863^▲▲^^*∗∗*^	13.92 ± 1.270^▲▲^^*∗∗*^
E	16.42 ± 1.234^▲^^*∗*^	3.42 ± 0.514^▲▲^^*∗*^	9.75 ± 1.162^▲▲^^*∗∗*^	14.50 ± 1.138^▲▲^^*∗∗*^^∆^
F	15.67 ± 1.047^▲^^*∗*^	4.08 ± 0.468^▲▲^^*∗*^	6.92 ± 0.657^▲▲^^*∗∗*^	11.50 ± 1.019^▲▲^^*∗∗*^

Group A is the control group (male rabbit without any treatment); Group B is the dry eye model rabbit group; Group C is the dry eye rabbit model group treated with 1 mg/ml *Buddleja officinalis* Maxim eye drops; Group D is the dry eye rabbit model group treated with 1.5 mg/ml *Buddleja officinalis* Maxim eye drops; Group E is the dry eye rabbit model group treated with 3 mg/ml *Buddleja officinalis* Maxim eye drops; Group F is the dry eye rabbit group treated with testosterone. ^▲▲^*P* < 0.01 compared with Group A; ^▲^*P* > 0.05 compared with Group A; ^*∗∗*^*P* < 0.01 compared with Group B; ^*∗*^*P* > 0.05 compared with Group B; ^∆∆^*P* < 0.01 compared with Group F; ^∆^*P* > 0.05 compared with Group F.

**Table 2:** Comparison of average optical density values of PI3K, Akt, and caspase-9 ($\bar{x}$ ± SD).

Group	*n*	PI3K	Akt	Caspase-9
A	12	0.4055 ± 0.0191^▲^	0.4715 ± 0.0177^▲^	0.3570 ± 0.0113^▲^
B	12	0.3379 ± 0.0445	0.3685 ± 0.0235	0.4074 ± 0.0264
C	12	0.3828 ± 0.0115^▲^^*∗*^	0.4023 ± 0.0214	0.3980 ± 0.0219
D	10	0.3807 ± 0.0106^▲^^*∗*^	0.4067 ± 0.0053	0.3572 ± 0.0097^▲^^*∗*^
E	12	0.4380 ± 0.0273	0.4662 ± 0.0461^▲^^*∗*^	0.3626 ± 0.0250^▲^^*∗*^
F	10	0.3918 ± 0.0177^*∗*^	0.4807 ± 0.0473^*∗*^	0.3516 ± 0.0273^*∗*^

^*∗*^ *P* > 0.05 compared with Group A; ^▲^*P* > 0.05 compared with Group F.

## Data Availability

The data used to support the findings of this study are available from the article, and the data are available and can be shared with the publisher upon request.
